# Essentials and Pertinence of Cold Plasma in Essential Oils, Metal–Organic Frameworks and Agriculture

**DOI:** 10.1002/fsn3.4583

**Published:** 2024-11-05

**Authors:** Mujahid Ameen Khan, Sumia Akram, Rabia Naeem, Muhammad Umair Kamal, Gulzar Muhammad, Muhammad Mushtaq, Farooq Anwar, Ahmad Hosseini‐Bandegharaei

**Affiliations:** ^1^ Department of Analytical Chemistry and Chemical Metallurgy, Faculty of Chemistry Wroclaw University of Science and Technology Wroclaw Poland; ^2^ Division of Science and Technology University of Education Lahore Pakistan; ^3^ Department of Chemistry Government College University Lahore Pakistan; ^4^ Deparment of Chemistry University of Agriculture Faisalabad Pakistan; ^5^ Department of Food Science, Faculty of Food Science and Technology Universiti Putra Malaysia Serdang Selangor Malaysia; ^6^ Institute of Chemistry University of Sargodha Sargodha Pakistan; ^7^ Faculty of Chemistry Semnan University Semnan Iran; ^8^ Department of Sustainable Engineering Saveetha School of Engineering, SIMATS Chennai Tamil Nadu India; ^9^ Centre of Research Impact and Outcome Chitkara University Rajpura Punjab India

**Keywords:** agriculture, cold atmospheric pressure plasma, essential oils, metal–organic frameworks

## Abstract

Cold atmospheric pressure plasma (CAPP) comprises an ensemble of ionized gas, neutral particles, and/or reactive species. Electricity is frequently used to produce CAPP via a variety of techniques, including plasma jets, corona discharges, dielectric barrier discharges, and glow discharges. The type and flow rates of the carrier gas(es), temperature, pressure, and vacuum can all be altered to control the desired properties of the CAPP. Since a few decades ago, CAPP has become a widely used technology with applications in every walk of life. The plasma activated liquid mediums like water, ethanol, and methanol have been merged as novel sterilizers. With recent advancements in material science, particularly work on metal–organic frameworks (MOFs), essential oils, and agricultural technologies, CAPP has become a vital component of these advancements. Likewise, CAPP has been found as a green and benign technology to induce early seed germination and plant development. This review covers the critical components of CAPP, the production of reactive oxygen and nitrogen species, and mechanisms by which CAPP‐based technologies are applied to agricultural products, MOFs, and essential oils.

## Graphical abstract

Cold atmospheric pressure plasma (CAPP) comes with high‐energy reactive species. The present monograph explains the workings of CAPP technologies, their salient features, pertinence to MOF synthesis, seed germination/sterilization, and production/preservation of essential oils.

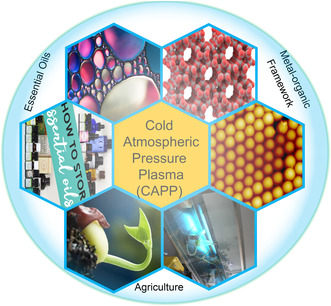

## 1 Introduction

Most commonly, ionized, neutral, and partially ionized species make up plasma, the fourth state of matter. Electrons, charged particles, neutral species, and radiations (heat) can all be found in natural plasma (Samukawa et al. 2012). According to a typical definition given by Kelley, Potter, and Liley (2022, September 5) https://www.britannica.com/science/plasma‐state‐of‐matter) the term “plasma” refers to the conducting medium produced by gases with an equal amount of cations and anion. Each electron is accountable for one negative charge, which is how the terms negative and positive charge are used to characterize excess and shortage of electrons. Electrons and cations are maintained apart in the elementary plasma (sun) by extremely high temperatures. Plasma states, aside from metallic crystals, are infrequently seen close to or at the earth's surface. To make plasma for various therapies and technological uses, we artificially create it in laboratories (Bárdos and Baránková 2010).

Since the majority of the materials melt at temperatures below 10^4^ K, no specific compartments can be constructed to make typical plasma using the current techniques. However, an electric field is frequently used to generate and accelerate free electrons from gaseous media. Once more, utilizing electrodes or the magnetic fields generated by transformers, the electric fields are applied. By applying these methods, temperatures up to 10^7^ K and electron densities of roughly 10^19^ m^−3^ have been achieved. Ionization occurs often in light‐induced plasma as a result of the flux of energetic photons. The energies produced by fusion reactions produce the solar and stellar plasma (Spitaleri et al. 2016). The alkali metals and certain gases in the atmosphere may ionize with the ultraviolet and/or x‐rays of sunlight. It is interesting to mention here that when the sun sets a certain portion of this plasma reverts back to a stable gaseous state, but ions like O^+^ are stable enough to live until the next sun rises (Keika, Kistler, and Brandt 2013).

The plasma has been frequently applied for the degradation and deactivation of harmful and environmentally hazardous compounds such as antibiotics (Cheng et al. 2022; Lou et al. 2012; Magureanu et al. 2011; Nguyen et al. 2021), and endocrine disrupting hormones (Dzimitrowicz, Motyka‐Pomagruk, et al. 2021) in wastewater, juices (Ozen and Singh 2020), milk, and related products (Rathod, Kahar, et al. 2021; Sharma 2020), seafood (Olatunde, Shiekh, and Benjakul 2021; Rathod, Ranveer, et al. 2021), essential oils (Sana et al. 2021), and rice (Misnal et al. 2021). Once treated with plasma, food products have been found resistant towards oxidation, free of various contaminants (Bourke et al. 2018, 2017; Saremnezhad et al. 2021), and become rich in antioxidant constituents such as phenolics (Keshavarzi et al. 2020). Conrads and Schmidt (2000) and Moisan and Zakrzewski (1991) are among the few commonly referenced authors who have attempted to summarize the benefits and drawbacks of plasma production and survival. Similarly, a plethora of research has been published to highlight the applications of plasma in medicine (Heinlin et al. 2011) and cancer treatment (Keidar 2015), environment and energy (Kim et al. 2016), synthesis and surface treatment (Bárdos and Baránková 2010), and energy storage and conversion (Bárdos and Baránková 2010). von Woedtke et al. (2013) have discussed the direct and indirect applications of plasma in medicine. However, the literature currently available does not provide comprehensive information regarding the essentials and pertinence of cold atmospheric plasma for essential oils, agriculture, and metal–organic frameworks (MOFs). This review can serve as a source of up‐to‐date methodical information on plasma classification, production systems, and chemistry. The review also emphasizes new developments and proposals pertaining to CAPP treatment of MOFs, agricultural operations, and essential oils.

## 2 Essentials of Plasma

**FIGURE 1:**
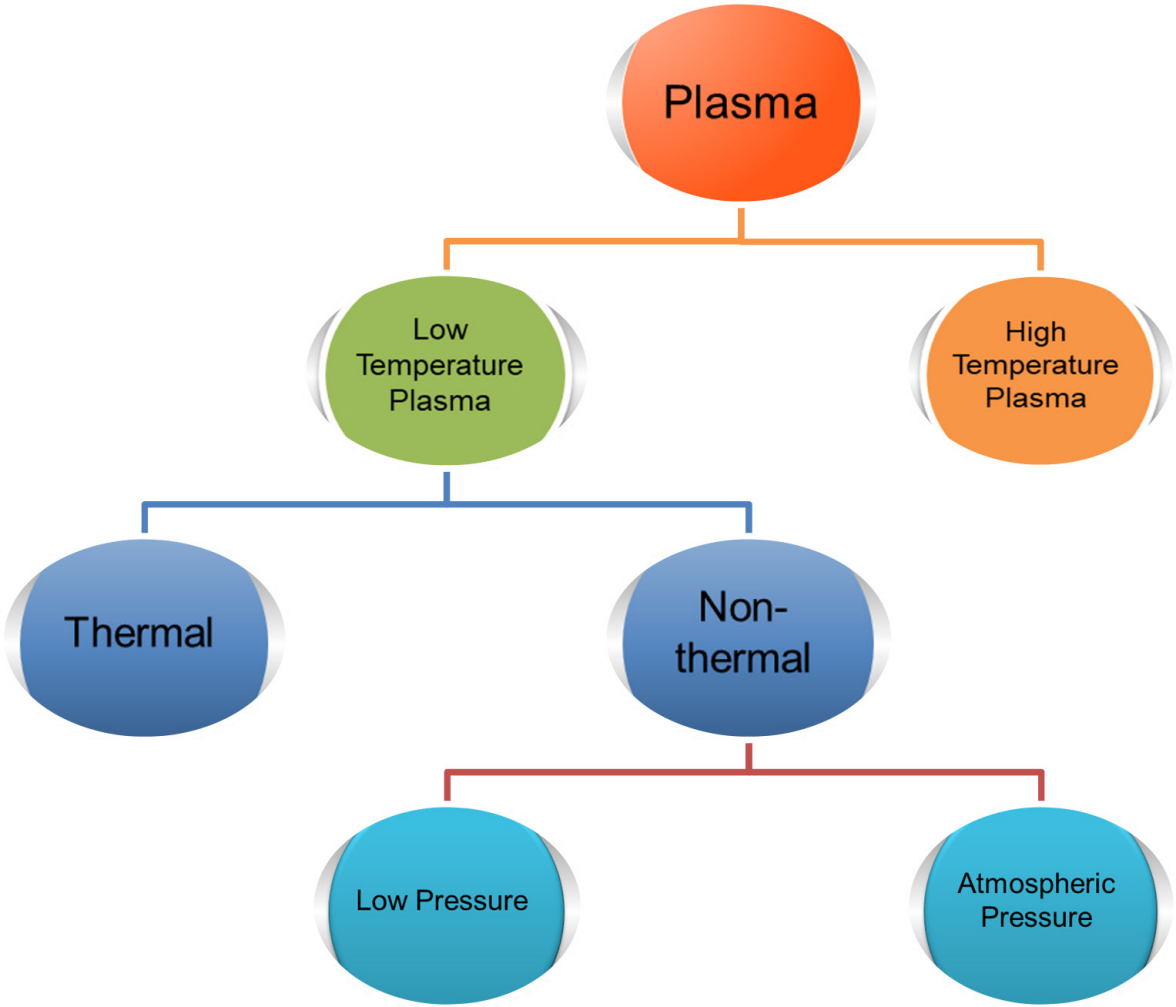
A generic illustration representing the plasma classification.

The important aspect of natural plasma to bother about is its temperature, that is, a superheated form of matter which exists at millions of K. The artificial plasma, on the other hand, can exist at low‐temperatures (Adamovich et al. 2017; Samukawa et al. 2012). The low‐temperature plasma, also known as cold plasma (CP), can either be produced via thermal or non‐thermal routes. The thermal plasma works at temperatures of several thousand K and is commonly used in Inductively coupled plasma (ICP) and related technologies for ionization, while the non‐thermal plasma (NTP) exits at room temperature (Adamovich et al. 2017; Laroussi et al. 2022), except in non‐equilibrium plasmas, where the electrons are often heated up to 30,000 K. The NTP is generally produced with the help of electric discharge, often referred to as “glow discharge,” either under vacuum (used as an ionization source in MS or OES, etc.) or at atmospheric pressure (touchable plasma or cold atmospheric plasma). The cold atmospheric pressure plasma (CAPP) has emerged as an eccentric tool for the food industry, biomedical, agricultural, catalysis, and material sciences (Domonkos et al. 2021; Xiong 2018). Besides, the use of CAPP in essential oil extraction, metal organic framework (MOF) synthesis, and agricultural preservation has now become a source of aspiration for scientists and researchers worldwide (Bárdos and Baránková 2010; Domonkos et al. 2021; Gorbanev, Privat‐Maldonado, and Bogaerts 2018; Sriraksha et al. 2022). The various types of plasma are mentioned in Figure 1.

### 2.1 RONS

**FIGURE 2:**
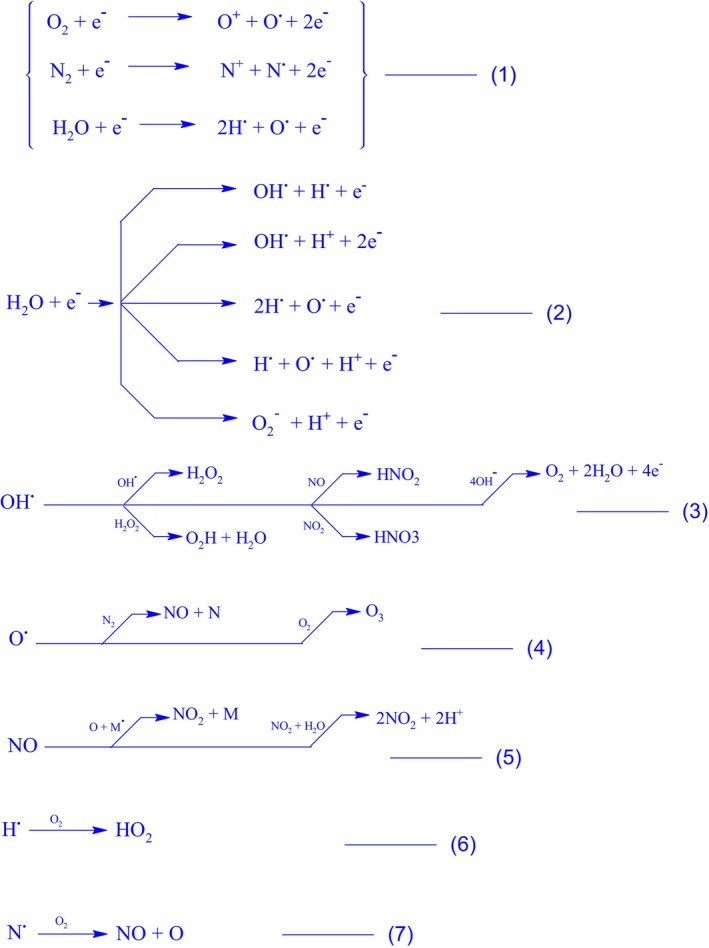
Scheme of reactions (1–7) involved in the production of reactive oxygen and nitrogen species (RONS).

The emerging trend in CAPP applications can be justified while considering the temperature of the system and the number of reactive species generally produced during the process, also referred to as reactive oxygen and nitrogen species (RONS) (Wende et al. 2019). The survival of CAPP at low temperature and atmospheric pressure has opened numerous opportunities and challenges regarding its use and application, and understanding the nature of RONS can help us further expand the range of its applications. Some of the species produced by the CAPP application are transient, such as radicals and/or excited species, While others are stable, such as neutral species. Reactive species' types and numbers depend on the energetics and nature of CAPP as well as the make‐up of the medium. The nature and quantity of reactive species vary with the nature and energetics of CAPP as well as the composition of the medium, but in most of the cases, The reaction scheme presented in Figure 2 describes the production of hydroxyl radicals (OH^·^), nitric oxide radicals (NO^·^), nitrogen dioxide (NOO^·^), peroxynitrite nitrogen trioxide (ONOO^·^), oxy (O^·^), superoxide anion radical (O_2_^·−^), and hydride radical (H^·^). The ions such as OH^−^, O^−^, O_2_^−^, NO^−^, N_2_^+^, ONO^−^, Peroxynitrite (ONOO^−^), Peroxynitrate (OONOO^−^), H_3_O^+^, and neutral species such as Hydrogen Peroxide (H_2_O_2_), Ozone (O_3_), O_2_, hydroperoxyl (O_2_H), NO_2_, NO_3_, and HNO_3_ have also been reported (Domonkos et al. 2021; Jamróz et al. 2014). Besides, many CAPP reactions may involve the production of atomic species such as H, O, N, and singlet oxygen (^1^O_2_).

Apart from these wondrous species shown in Figure 2, high‐energy electrons (*e*_eq_) and radiations across the spectrum, especially Ultraviolet (UV) radiations, along with a strong electric field, are generated during plasma treatment (Gorbanev, Privat‐Maldonado, and Bogaerts 2018; Weidinger and Kozlov 2015). The majority of the reactive species are produced simultaneously inside the plasma arc or in the medium being treated with the CAPP (Bruggeman et al. 2016). The RONS can initiate a wide range of chemical reactions (Pai et al. 2018; Šimončicová et al. 2019), inhibit the production of human melanoma cells (Terefinko et al. 2021; Yadav et al. 2020), facilitate the surface treatment (adhesion, maceration, biofilm formation, sterilization, and activation) (Tabares and Junkar 2021), and decontaminate wastewater and/or air streams (Hashim et al. 2016).

The nature and quantity of RONS actually produced during the CAPP application decide the application and usefulness of this technology (Gorbanev, Privat‐Maldonado, and Bogaerts 2018). Furthermore, the nature and quantity of RONS produced in plasma or medium during application can alter the chemical composition, physicochemical, functional, bioactive, textural, and sensory attributes, and shelf life of the final food or pharmaceutical product (Sruthi et al. 2022). In addition to the power, voltage, and design of the plasma tube, career gas, and type of electrodes affect the concentration of RONS produced.

### 2.2 RONS Characterization

The analytical techniques that are used to determine the RONS produced during CAPP treatment include but are not limited to UV–visible spectroscopy, Optical emission spectroscopy (OES), fluorimetry, colorimetry, and high‐performance liquid chromatography, coupled with various detectors such as TCD, DAD, MS, and electron spin resolution (ESR) spectroscopy (Greda et al. 2016; Tendero et al. 2006). Furthermore, the utilization of radiotracers, that is, ^18^O and ^13^N, can also help to determine the origin, interactions, and fate of RONS (Wende et al. 2020). It has been observed that during the analysis of short‐and long‐lived species, several other opportunities or situations are missed intentionally or unintentionally. For example, various methods can establish the presence of hydroxyl radical (one of the most important RONS); however, it is often ignored that the hydroxyl radical can be formed from H_2_O_2_ and/or can add up to make H_2_O_2_, having a direct relation with H_2_O_2_ concentration. Several authors have highlighted the situation, uncertainties, limitations, and side reactions that need to be addressed while studying RONS species (Greda et al. 2016; Pankaj and Keener 2017; Samukawa et al. 2012; Zhou et al. 2020).

## 3 CAPP Applications

**FIGURE 3:**
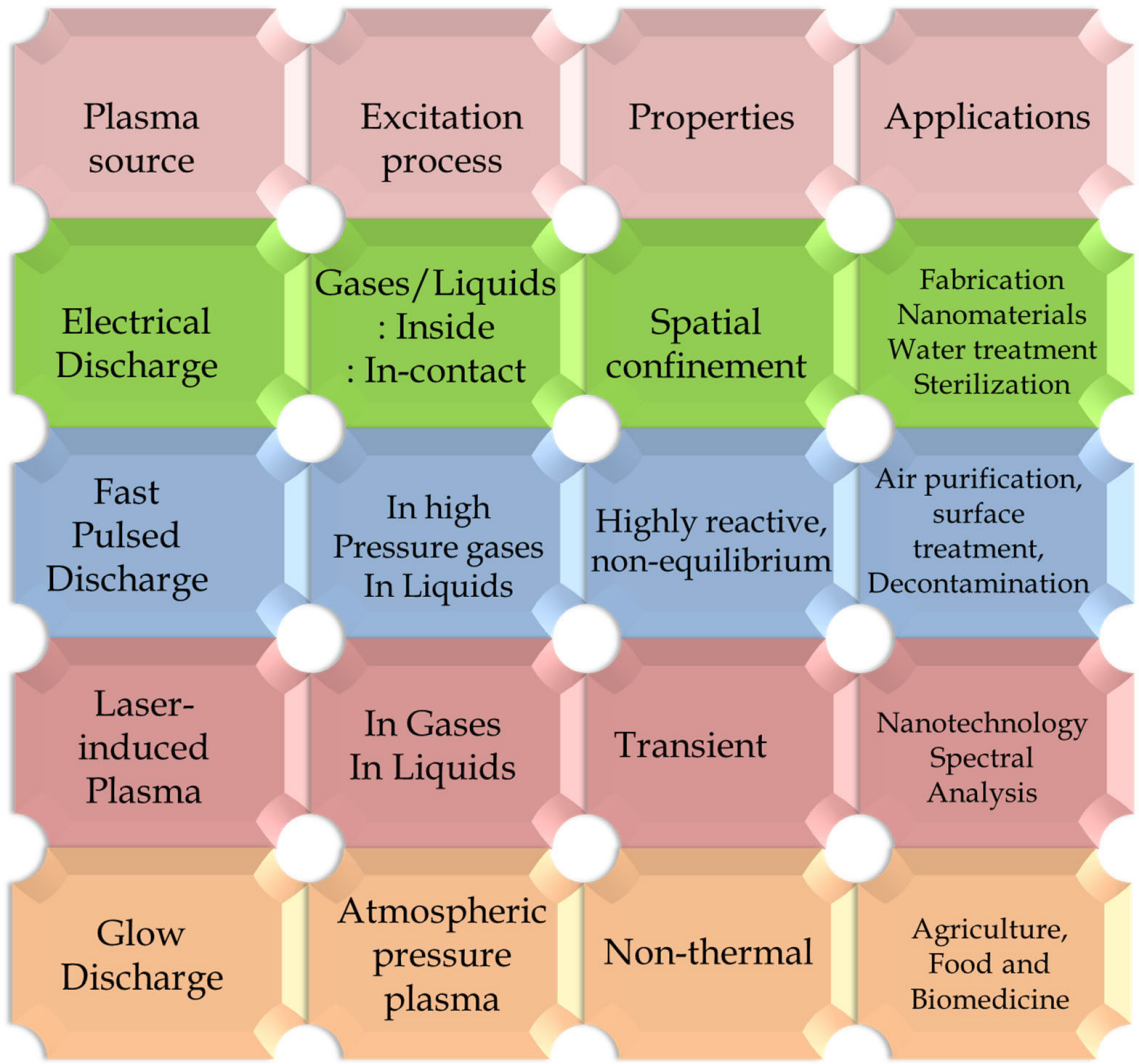
Illustration for Plasma generation, processes, properties, and major RONS‐driven applications (Adamovich et al. 2022).

Plasma offers an enormous cascade of uses owing to the presence of RONS species, which are responsible for its exceptional sterilizing capacity, catalytic, reducing potential, and degradative properties. The reducing potential of RONS has opened a wide range of new opportunities for the synthesis of nanomaterials (NMs). The oxidizing power of RONS free radicals has been exploited for water purification and remediation (Barjasteh et al. 2021), decontamination of waterborne pollutants (Hijosa‐Valsero et al. 2014), and production of plasma‐activated water (Ki et al. 2020; Zhou et al. 2020). RONS has bactericidal potential against many bacterial species (Lotfy 2020; Pankaj et al. 2017; Yang, Chung, et al. 2021), including antibiotic‐resistant bacterial species (Yang, Niyazi, et al. 2021), when applied directly or via the synthesis of specialized materials (Yang, Chung, et al. 2021). In these contexts, Figure 3 summaries the role of various plasma sources in the synthesis of nanomaterials (Chen, Li, and Li 2015; Saman, Ahmad, and Buntat 2021; Sohn and Murali 2021; Vinoth Kumar et al. 2021; Woodard et al. 2018), catalytic stabilization (Cyganowski et al. 2019, 2021; Tyczkowski and Kapica 2007), biomedical (Chen et al. 2022; Dubey et al. 2022), and agricultural science (Chen and Thimsen 2020; Dzimitrowicz et al. 2018; Mariotti et al. 2012; Motyka‐Pomagruk et al. 2021; Roy et al. 2021).

## 4 Essentials of CAPP Production

In this section, we will highlight the essentials of various methods capable of producing CAPP, mainly the operating principles and working parameters. These designs may help the readers to speculate about the subsequent use, nature of the interaction, RONS species, and their interaction with target surfaces, that is, solids, liquids, and/or gases (Qiang Chen, Li, and Li 2015; Greda et al. 2016; Mariotti et al. 2012).

### 4.1 Dielectric Barrier Discharge

**FIGURE 4:**
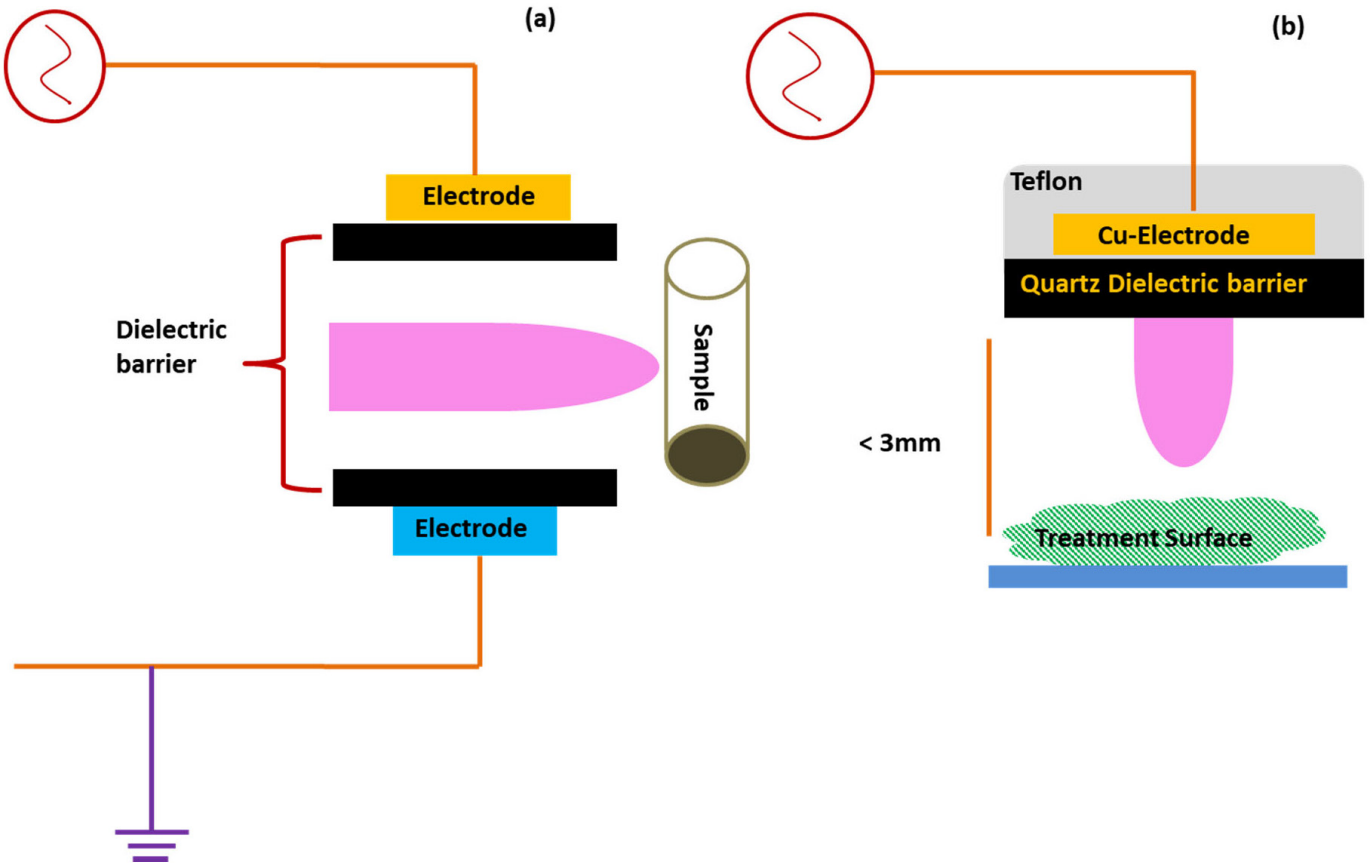
Typical construction of conventional DBD (a) in which electrodes are separated by some dielectric and (b) a floating electrode type DBD where the second electrode is not grounded.

The Dielectric Barrier Discharge (DBD) usually comes with two flat metal electrodes separated by some insulating dielectric material. One of the electrodes is kept at a high potential while other works as a grounded electrode (Figure 4a), Once potential is applied, the carrier gas between the electrodes receives electrical discharge to produce the plasma. The alternating current (AC) in the kHZ range can produce electrical discharge, and the process involves the consumption of 10–100 watts of electrical energy (Hoffmann, Berganza, and Zhang 2013). The dielectric barriers can be made up of glass, quartz, ceramics, mica, polymers, and semiconductors like gallium arsenide, which can make DBD work with direct current (DC). Dielectric barrier discharge‐based plasma systems, usually referred to as “silent plasma” (Kogelschatz 1990), are one of the most prevalent devices and usually operated as a stationary mode of CAPP treatment. In DBD systems (Figure 4a), one or both electrodes are covered with dielectric materials (both planer and cylindrical models), and the system creates plasma using an HV power source corresponding to the frequency in kHz (Laroussi and Akan 2007). Besides, arcing does not occur because here the discharge current does not increase continuously (which may lead to arcing). The ions/charges produced are extinguished by the dielectric materials, which causes a voltage drop during charge collection when plasma is ignited. Divya Deepak et al. (2017) constructed a DBD‐based atmospheric pressure plasma jet and found that, in addition to electrode configuration, the discharge gas also plays a significant role in the low‐power operation of the cold plasma jet. During DBD‐based plasma ignition, instead of a fine arc, multiple cylindrical plasma streamers are observed randomly for very short instances (i.e., nanoseconds) between the electrodes (Kogelschatz 1990, 2003; Laroussi and Akan 2007). However, DBD is able to produce streamer‐free diffused plasma with the optimum current frequency range and gas flow rates (Laroussi 2002; Massines et al. 1998).

The DBD‐type CAPP system operates in many configurations, like coaxial (Figure 4a), floating electrode (Figure 4b), planar, and parallel plates. Another modification is known as “piezoelectric barrier discharge,” in which high voltage is generated from a piezo‐transformer. The piezo‐electric materials like lead zirconate titanate, when operated far from the ground electrode, also produce corona discharge on the sharp edges of the piezo‐transformer. Interestingly, piezoelectric barrier discharge, for its compact design and construction principle, works as a more economical source of plasma.

In DBD with a double layer of dielectric barriers, the plasma and reactants are isolated from electrodes. This configuration can avoid electrode etching and contamination caused by plasma, but at the same time it produces a smaller number of stronger electrical pulses. Meanwhile, many modifications in double dielectric barrier discharge (DDBD) have appeared to be more efficient non‐thermal plasma sources. For example, Li et al. (2022) constructed co‐axial DDBD comprising two layers of glass dielectric, a high voltage electrode, and a ground electrode and found that the discharge intensity of the inner tube is much stronger than that of the outer tube.

The DBD or floating electrode finds applications in surface treatment, sterilization, organic synthesis, nanomaterials, and excimer formation (Adamovich et al. 2017; Bárdos and Baránková 2010; Bruggeman et al. 2016). Besides, apart from configuration and principles, DBD working parameters, which could include voltage applied, type of electrodes, carrier or working gas(es), flow rates of gas(es) or liquid(s), current frequency, treatment distance, and time, have a particular impact on the kind, concentration, and behavior of reactive species produced as well as the overall impact of plasma treatment. These CAPP devices need continuous sine waves and work at high ignition voltage (1–10 kV).

### 4.2 Corona Discharge

**FIGURE 5:**
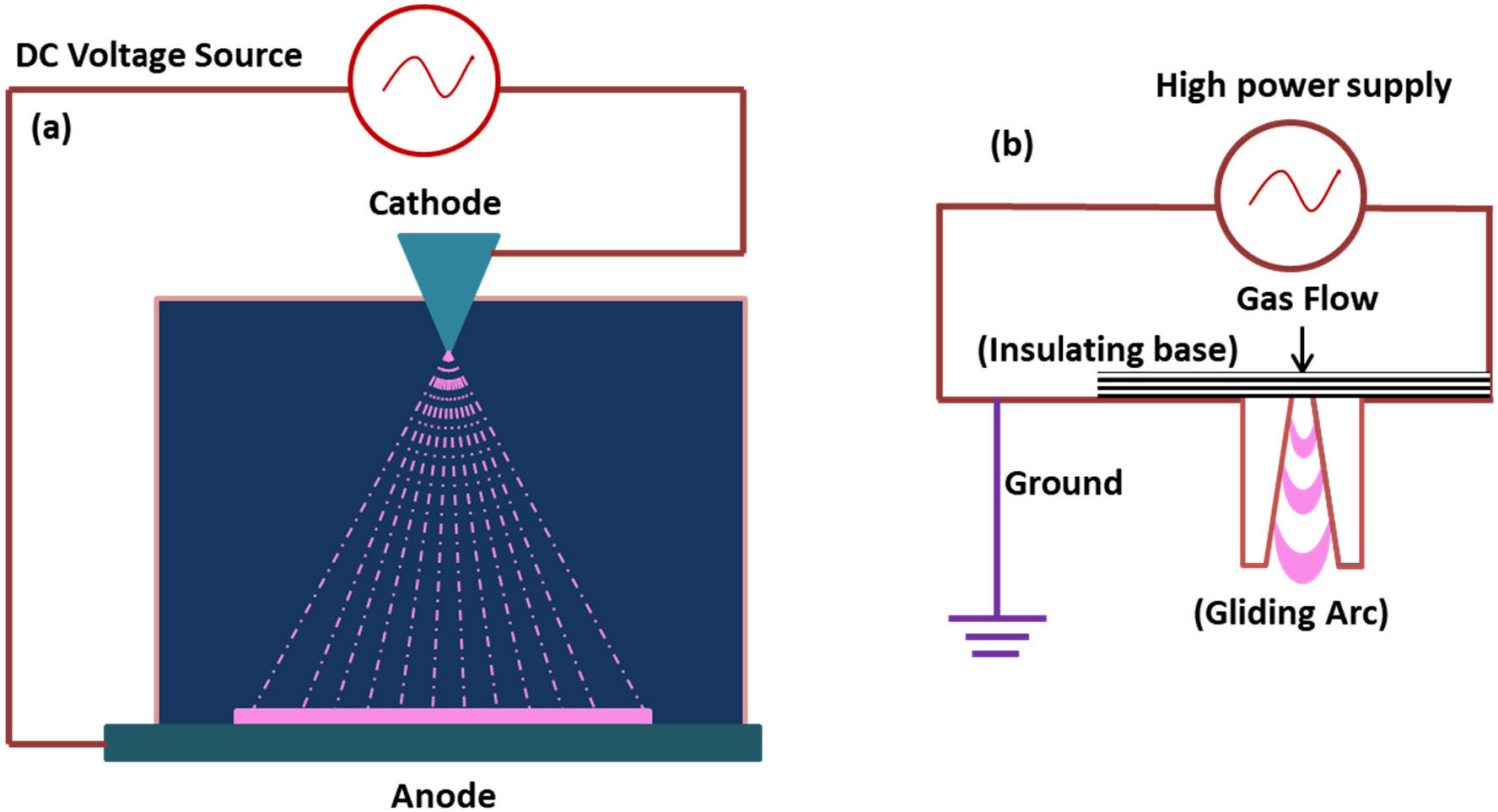
Schematic representation of corona discharge (a) and Gliding Arc Discharge (b).

The Corona Discharge (CD), also known as Townsend discharge (Figure 5a) covers the ionization processes where electrons from the cathode (negative CD) accelerate towards the anode and ionize the gas molecules. On the other hand, the gas surrounding the high‐voltage electrode (anode) may ionize (positive CD), and these electrons may propagate towards the anode. In both cases, a uniform plasma glows around the sharp edges of electrodes. The CD produces low current (1–100 μA) and discharge power at higher voltage; however, the key advantage regarding the use of CD lies in the simplicity of the direct current source. CD often happens as an unwanted phenomenon in high‐voltage appliances because it occurs in place around an electric field where the strength of the electric field exceeds the dielectric constant of air or medium. In addition to CO_3_^−1^, O_2_, NO_3_^−1^, and N_2_O, a mixture of SF_6_, its mixture with water and Freon 113, and C_3_F_6_ has also been found in CD produced from pure gas and/or air (Skalny et al. 2004). This type of ionization process occurs in devices like lasers, air conditioners, photocopiers, etc.

### 4.3 Gliding Arc Discharge

The Gliding Arc Discharge (GAD) stands as a unique type of non‐equilibrium NTP that have high power, plasma density, electron temperature, selectivity, and operating pressure as compared to other non‐equilibrium NTPs (Lie et al. 2006). The GAD consists of two identical metal electrodes a few millimeters apart (Figure 5b). In this approach, the arch initiates at the shortest distance between two electrodes and elongates due to transverse gas flow. At the initial point, the current is at its maximum while the voltage is at its minimum and more power is required to sustain the arc at its maximum power supply. The GAD has the ability to produce both thermal and non‐thermal plasma at the various regions of electrodes. However, the highly variable nature of the discharge makes it unsuitable for controlled experiments. Besides, these techniques provide an easy opportunity to produce hard oxidation, excellent surface treatment, chemical degradation, and bacterial decontamination of‐products. The GAD has been frequently adopted in the devices used to decontaminate air and water samples.

### 4.4 Atmospheric Pressure Plasma Jet

**FIGURE 6:**
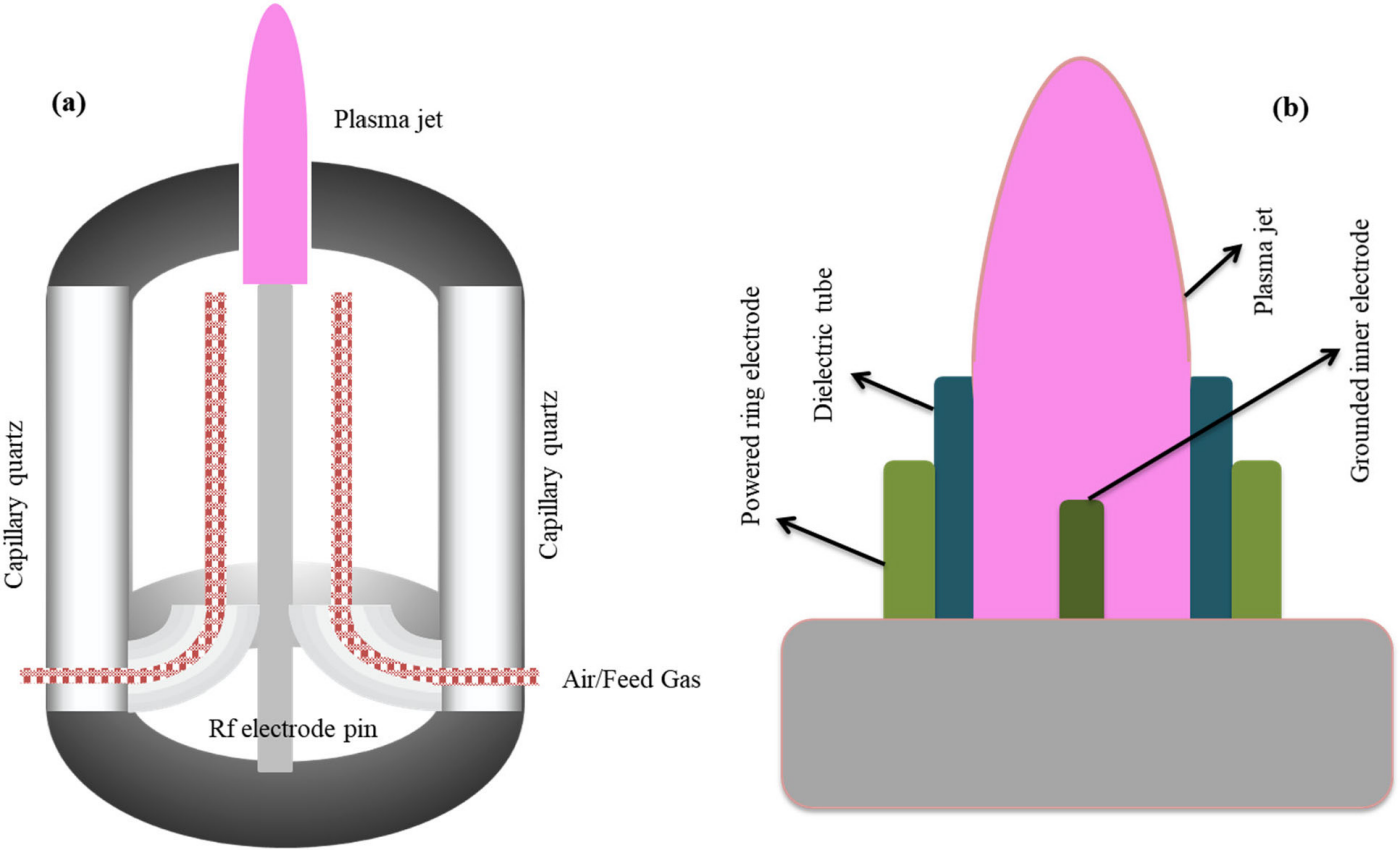
A process diagram for two different variants of atmospheric pressure plasma jets suggested by Weltmann et al. (2009) (a) and Jnido, Ohms, and Viöl (2019) (b).

**FIGURE 7:**
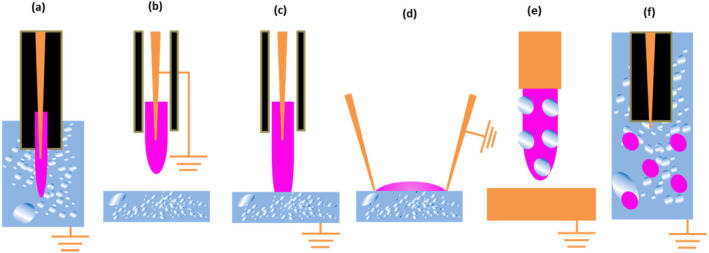
Demonstration of plasma impact patterns explained by Bruggeman et al. (2016): Direct discharge into liquid medium (a), non‐contact plasma treatment (b), Gaseous plasma with liquid electrode (c), surface discharge (d), plasma with dispersed liquid (e), and discharge bubbles. Orange, purple, blue, and black colors represent electrode, plasma, medium, and dielectric, respectively.

One of the most often used plasma reactors for producing CAPP species is the atmospheric pressure plasma jet (APPJ) device (Figure 6). This system has two electrodes, the high‐energy electrode and the ground electrode, just as in previous electrically induced plasma systems. The former is attached to a radio frequency (RF) or high voltage (HV) power source. In the region between these two electrodes, the carrier or working gas(es) ionizes, producing the distinctive RONS. Lu et al. (2016) have comprehensively reviewed the formation, transport, and biological application of reactive species making up non‐thermal atmospheric plasma. A schematic illustration of these species has also been drawn in Figure 2. These species involve reactive electron, ionic, atomic, and radical species of auxiliary gases. A wide range of theoretical models has been presented and applied to explain the transportation of these species from the point of generation to interaction with medium. These authors have also reviewed the fundamental physics for the formation and propagation of ionization waves. According to these authors, the ionization waves which appear in various types of plasma applications, such as corona discharge and surface and volume‐based dielectric barriers, are responsible for the pre‐breakdown gases. Besides, the ionization waves can have various spatial structures, as shown in Figure 7. The plasma bullets formed in the APPJ may offer many interesting physical effects as compared to other ionization techniques (Lu et al. 2014). One nozzle‐like opening in the apparatus generates a plasma output that resembles a needle. Important variables impacting the formation of RONS and their interactions include the type of electrode, the distance between electrodes, gas flow rate, applied voltage or frequency, and treatment time (Domonkos et al. 2021; Hoffmann, Berganza, and Zhang, 2013). Deepak, Joshi, and Prakash (2018) have attempted the electrical characterization of a cold plasma jet under pin configuration. The applied voltage, which has a significant impact on the chemistry and processes of the plasma, is one of the most crucial variables. Numerous studies examined the relationship between the voltage applied, the amount of reactive species present, and the function (degradation, decontamination, deactivation, etc.) of the plasma. The concentration of reactive species has often been shown to be directly correlated with applied voltage (Förster, Mohr, and Viöl 2005). In another study, Divya Deepak et al. (2016) constructed a cold plasma jet based on a double‐ring electrode configuration. The setup was tested over a wide range of power supply frequencies and voltages and found that peak power consumed for glow discharge operation was 1.27 W.

One of the earliest versions of APPJ was developed by Koinuma et al. (1992), which has been further modified by Stoffels et al. (2002), Laroussi and Lu (2005) and Weltmann et al. (2009). Figure 6a,b describes the design of such APPJ systems named “plasma needle” and “plasma pencil.” Several other developments of APPJ are under consideration by many research groups (Foster et al. 2010; Sun et al. 2006). The efficiency of APPJ systems varies depending on the current kind, such as pulsed, jet, etc. Direct current (DC) or alternating current (AC) can be used to power them (AC) (Walsh, Shi, and Kong 2006a, 2006b). They can have a tubular (hollow) or cylindrical shape, or they can be based on the DBD system (Chen et al. 2006; Cheng, Liye, and Zhan 2006; Teschke et al. 2005). The following designs were included in certain plasma jet systems:

1. One of the straightforward designs involves two planer electrodes inside a hollow Teflon or quartz tube. Carrier gas is allowed to flow through the hollow tube, creating a plasma jet at one of the tube's ends and claimed as a “plasma brush.” This system is reported to be operated under continuous as well as pulsed DC with gas flow rates at 3500 and 1000 scm, respectively (Duan, Huang, and Yu 2005).
2. Plasma jet system based on DBD design, having an HV electrode made of brass circled around the cylindrical dielectric barrier (Figure 6b) with a brass ground electrode in the middle of the dielectric cylinder. In this system, carrier gas argon was used at a flow rate of 250 L h^−1^ and an applied voltage of 25 kV (pulsed repetition at 25 kHz) to generate a plasma jet. The plasma generated is claimed to be touchable and can help increase the wood wettability (Förster, Mohr, and Viöl 2005).
3. Another DBD‐based plasma jet consisted of a dielectric barrier made of quartz capillary in which the inner electrode (tungsten wire or rod) is connected to the HV battery (Figure 6a) while the outer brass electrode wrapped around the dielectric is grounded. In this system, Ar, He, or N_2_ can be used as carrier gases at an applied voltage of 1–1 5 kV (pulsed repetition at 15 kHz). The researchers found that the velocity of carrier gas in order to generate the plasma jet is critical. And according to another study, He, Ar, and N_2_, the critical velocities beyond which the plasma jet can be created are 3, 5, and 8 ms^−1^, respectively (Zhang et al. 2006).
4. Walsh, Shi, and Kong (2006a, 2006b) evaluated two distinct power sources for DBD‐based plasma jet systems, namely sinusoidal and pulsed power sources. An HV electrode was wrapped around the dielectric tube as part of the plasma jet's basic construction, and a ground electrode was placed some distance away (3–5 cm) from the dielectric. He was allowed to flow down an empty tube at a rate of 5 L min^−1^, resulting in the creation of jet plasma between the electrodes. For sinusoidal stimulation, an applied voltage of 7.3 kV at a frequency of 7 kHz was employed. The pulsed power source, however, employed 71‐μs‐wide pulses with an HV of 4 kV. It was determined that a pulsed power source was more efficient than a sinusoidal one from an energy standpoint because it would require 12 times as much energy to produce the same quantity of atomic oxygen, a crucial reactive species for activities that involve surface oxidation.
5. One of the smallest jet systems, termed “plasma pencil” by the authors, has a different design than previous ones. In this design, two electrodes (copper wires) are attached to a disk (perforated alumina) with a central cylindrical hole (3 mm) and inserted in a dielectric cylindrical tube. These electrodes are connected to an HV power source capable of producing pulsed plasma at power up to 10 kV (at the repetition of 10 kHz) with a 60 ns time gap between the rise and fall of pulses (Laroussi and Lu 2005).
6. Another important plasma jet system termed “atmospheric pressure glow discharge” (APGD)—either with flowing liquid cathode (FLC) or flowing liquid anode (FLA) using a DC have been designed and widely used in which continuous flow of liquid is intended (Cyganowski et al. 2019, 2021; Dzimitrowicz et al. 2018). One such system is designed and patented by Dzimitrowicz, Jamroz, et al. (2021). A sharpened tungsten electrode (anode) and a quartz‐graphite electrode (cathode) are arranged vertically in this setup. With the aid of Pt wire, this quartz‐graphite electrode—which serves as an FLC or FLA—is integrated into the electronic circuit. The anode is connected to an HV power source, which can produce voltages of up to 1400 V between them, and a current of 45 mA is maintained by a resistor that is part of the circuit. Either FLC‐dc‐APGD or FLA‐dc‐APGD are terms used to describe the entire systematics (Motyka‐Pomagruk et al. 2021).

Plasma is often created by APPJ‐based devices as a conical‐shaped beam that can only be used to treat a part of the samples. One of the core characteristics of these systems is the constant flow of liquid samples. Additionally, the type of sample, the precise area of the gas or liquid sample (specific point of contact/plasma‐liquid interface), or the atmospheric air, as well as the continuity, velocity, or flow rate of the liquid, gas, or air between electrodes, all affect the RONS produced by these devices. APPJ‐based systems create plasma, which is usually a conical‐shaped beam and able to treat a specific portion of the samples. In fact, the continuous flow of liquid samples is also one of the fundamental attributes of these systems. Furthermore, RONS produced by these systems is dependent on the kind of sample, a specific portion of gas/liquid sample (specific position of contact/plasma‐liquid interface) or the atmospheric air, and continuity/velocity/flow rate of the liquid, gas, or air between electrodes. Djemaa et al. (2020) investigated the impact of voltage on the sterilization process against some bacterial species and found that the higher voltage, producing more reactive species, offers better sterilization results. Another crucial element affecting the kinds and concentration of RONS species is the working or carrier gas (Sharma and Singh 2022).

The most often employed carrier gases are ambient air, argon (Ar), oxygen (O_2_), hydrogen (H_2_), nitrogen (N_2_), and/or their mixtures (Lotfy 2020). Researchers have found that a mixture of carrier/working gases, that is, N_2_ and a mixture of N_2_–O_2_, had a more detrimental impact on skim milk than using just nitrogen (Lotfy 2020; Sharma and Singh 2022). Besides, the plasma and material contact patterns as shown in Figure 7a–f affect the production of RONS and treatment effectiveness. In this figure, orange, purple, blue, and black colors represent electrode, plasma, medium, and dielectric, respectively. Likewise, carrier gas flow rates, treatment time also determine the outcome of the plasma‐contact interactions (Aadim et al. 2020; Sharma and Singh 2022). According to a report, the plasma therapy will result in a larger concentration of RONS if applied for prolonged period with increased flow rates (Aadim et al. 2020).

## 5 CAPP Activated Liquids

The exposure of liquid to either type of plasma can make it a good source of reactive oxygen and reactive nitrogen species and suitable for a variety of applications in food and agriculture. The Plasma activated liquids (PALs) are more frequently produced through DBD, Plasma jet, or CD and subsequently used as disinfectants in agriculture, preservatives in food science, and sterilizers in medical sciences. The actual physical and chemical properties of PAL depend upon the nature of the liquid under irradiation, irradiation time, and configuration/electrical properties of the plasma system. PAL has emerged as a promising technology with diverse applications in sterilization, decontamination, and preservation. The interaction between plasma and liquids has shown significant potential in various fields such as material processing, nanoscience, environmental remediation, and biomedicine (Rezaei et al. 2019). This technology has been utilized for surface decontamination, where active species generated in plasma devices are employed to decontaminate chemical agents and their surrogates (Li et al. 2013).

In general, the interaction of plasma with liquids, as shown in Figure 7, can initiate a series of chemical reactions leading to the formation of RONS. Under some special cases, the plasma gases may react with liquids present at the interphase; on the other hand, discharge taking place inside the liquid surface may initiate the breakdown of liquid molecules. For example, according to the reaction shown in Figure 2, the plasma activated water (PAW) may become rich in ^•^H, ^•^O, ^•^OH, and H_2_O_2_. Besides, HNO_2_/NO_2_^−^ and HNO_3_/NO_3_^−^ are the most abundant reactive species in PAW, as manifested in chemical reactions 5–10 of the scheme shown in Figure 2. The interaction between plasma and liquids involves the solvation of RONS produced by plasma into the liquid, thereby chemically activating the liquid (Meyer et al. 2023). Strategies to enhance this interaction include producing plasma directly in the liquid (Oinuma et al. 2020). Studies have shown that plasma‐activated liquids can be utilized for sterilization purposes in various settings, including medical facilities and research laboratories. For instance, hydrogen peroxide gas plasma sterilization has been widely adopted in hospitals for sterilizing medical equipment due to its efficacy and safety profile (Lee et al. 2007). The interest in PAW has been on the rise, with its applications expanding to biological sciences, medical fields, and the agriculture and food industry (Bradu et al. 2020). It should be mentioned here that RONS play a vital role in the subsequent application of plasma as a sterilizer, preservative, and/or other process. More detail regarding the function of PAW can be better understood in the following section covering the functions of RONS (Zhao et al. 2020). Likewise, the plasma activated ethanol solution (PAES) becomes rich in NO_2_^−^, NO_3_^−^, acetic acid, paracetic acid, and H_2_O_2_. The PAES works more efficiently to decontaminate microbial loads as compared to conventional ethanol. The higher sporicidal efficiency of PAW or PAES can be positively correlated with the concentration of RONS (Li et al. 2023).

## 6 Function of Reactive Oxygen and Nitrogen Species

As was already mentioned, CAPP produces a number of reactive species (i.e., RONS) when exposed to electric current. The type of RONS species, voltage or frequency used, type and flow rate of the carrier gas(es) and liquid(s), treatment period, etc., all affect the effectiveness of treatment, intensity, life span, and relative interactions. However, whether the target sample is dry or aqueous determines how these RONS interact and behave chemically (Judée et al. 2018). Excitation, deexcitation, ionization, disassociation, fragmentation, and several other types of reactions have been seen in the gas phase (and possibly also in the liquid phase) after CAPP treatment (Thirumdas, Kadam, and Annapure 2017).

The RONS species can be divided into different groups according to a wide range of characteristics, including their main component (e.g., ROS or RNS), shorter or longer life span, charged or neutral or radical, and their production in regions of plasma, gases, liquids, or solids. However, the most practical way to distinguish between these species is the type of species formed in relation to their period of formation, that is, primary, secondary, or tertiary reactive species. The collisions between neutrals and electrons, as well as the energy involved in these collisions, are what produce RONS (Barjasteh et al. 2021; Lamichhane et al. 2020). Primary reactive species (PRSs) are the instantaneously generated gas atoms (atomic H, N, O, etc.), molecules (NO, O_2_*^−^, etc.), ionized (M+), and excited (M*) neutrals as a result of these collisions (Lim et al. 2020; Lu et al. 2019).

Shorter lifetimes and high plasma intensity characterize these PRSs. For instance, it is stated that the lifetime of OH radicals is in the range of 2.7–1.2 μs, that of NO is 1.4 μs, and that of O_2_*– is 1.3 μs. Secondary reactive species (SRSs), such as H_2_O_2_, NO_2_, NO_3_, and O_3_, are created by recombination between comparable species in the ideal environment and PRSs (Lim et al. 2020; Lu et al. 2019). After dissolving or interacting with the liquid phase or target sample, secondary reactive species (SRSs) in the gas phase give rise to tertiary reactive species (TRSs), which have a relatively long lifetime (from a few milliseconds to some days) (Attri et al. 2015). PRSs undergo a transformation via recombination with other similar species in the optimal environment to form secondary reactive species (SRSs) such as H_2_O_2_, NO_2_, NO_3_, and O_3_ (Ghimire et al. 2018). SRSs in the gas phase give rise to tertiary reactive species (TRSs) after dissolving or interacting in the liquid phase or target sample, which have a relatively longer lifetime (from a few milliseconds to some days) (Kondeti et al. 2018; Lu et al. 2019). When dissolved in water, the pH of water decreases drastically up to 2 because H_2_O_2_, NO_2_, and NO_3_ are soluble in water, with NO_2_ and NO_3_ transforming into NO_2_^−^ and NO_3_^−^ respectively. After dissolving or interacting with the liquid phase or target sample, secondary reactive species (SRSs) in the gas phase give rise to tertiary reactive species (TRSs), which have a relatively long lifetime (from a few milliseconds to some days) (Barjasteh et al. 2021; Judée et al. 2018).

The physical and chemical reactions that occur as soon as CAPP comes into contact with water result in the production of OH radicals (both in gaseous and aqueous phases) (Attri et al. 2015; Barjasteh et al. 2021; Wende et al. 2019). The formation of OH radicals is a result of water dissociation either by electron collisions or collision with already excited particles or UV photolysis (cleavage of water). The researchers explained the various methods of formation of RONS, including OH radicals, H_2_O_2_, O_3_, NOx etc., and their interactions (Figure 7) in the following manner as a subsequent of CAPP‐initiated processes (Attri et al. 2015; Conrads and Schmidt 2000; Yepez et al. 2022).

Sharma and Rokita (2013) suggested that equation 2 is the most likely and practical reaction for the production of OH or H_2_O, as shown by the higher level of electrons detected in the liquid solution. The OH radical has a very high redox potential of 2.81 V, which rises with decreasing pH. The measurement of OH radicals has become a challenge because of their interconversions and the shorter duration of OH radicals (i.e., 2.7–1.2 μs). Overall, OH radicals are considered to be essential for decomposition, sterilization, cancer therapy, wound healing, and other processes (Graves 2014; Kaushik et al. 2019; Liu et al. 2014; Sarangapani et al. 2016). The degradative, reductive, oxidative, and decontaminative potential of CAPP has been extensively studied. Utilizing either direct or indirect methods, the authors tried to identify and quantify OH radicals utilizing analytical techniques like optical spectroscopy, electron spin resonance (ESR), and liquid chromatography‐mass spectrometry (Aggelopoulos, Hatzisymeon, et al. 2020; Aggelopoulos, Meropoulis, et al. 2020; Cheng et al. 2022; Fang et al. 2022; Gorbanev, Privat‐Maldonado, and Bogaerts 2018; Hatzisymeon et al. 2021; Magureanu et al. 2010; Marković et al. 2015; Tang et al. 2018; Xue et al. 2021).

By decomposing pesticides Sarangapani et al. (2016) documented the importance of OH radicals in the purification of water. Numerous other research teams have examined the importance of ROS, particularly OH radicals, in the creation of plasma‐activated water (PAW) with bactericidal capabilities and for residential water supply (Kaushik et al. 2019; Nguyen et al. 2019; Oehmigen et al. 2010). Significant biological consequences of OH radicals include bacterial inactivation, sterilization/disinfection, cell death, etc. The OH radicals also work as precursors for the production of H_2_O_2_ (Golkowski et al. 2012; Gorbanev, O'Connell, and Chechik 2016; Gorbanev, Privat‐Maldonado, and Bogaerts 2018; Xu et al. 2015).

Using a helium (He) based plasma jet system, Chauvin et al. (2017) examined the formation of OH radicals and their identification using electron paramagnetic resonance spectroscopy (EPR)/spin trapping with the aid of 5,5‐dimethyl‐1‐pyrroline N‐oxide (DMPO). In spin trapping, a chemical reagent is permitted to interact with short‐lived species like radicals to create an adduct (with a longer life span), whose concentration is determined using electron spin resonance spectroscopy, or EPR. For this, a DMPO aqueous solution is treated with jet plasma, resulting in the formation of the adduct DMPO‐OH, which is then detected using spin signals (Chauvin et al. 2017; Gorbanev, O'Connell, and Chechik 2016). They also demonstrated that OH radicals originate in the plasma and interact with other species to form other species, such as H_2_O_2_, H_2_O, O, and H. These measurement techniques do, however, have considerable uncertainties because of some adverse effects. The formation, interaction, and termination of OH radicals are said to be greatly influenced by the H_2_O_2_ concentration. On the contrary, Shiraki, Ishibashi, and Takeuchi (2016) used a chemical probe called sodium terephthalate (NaTA) or 2‐hydroxy terephthalic acid (HTA) to create and quantitatively evaluate the concentration of OH radical. In this experiment, OH radicals were produced in a NaTA solution using a pulsed plasma reactor, which changed the original NaTA into HTA, whose concentration was then determined by fluorometry. This work accurately quantified OH radicals by thoroughly analyzing and removing the influence and interference of other species like H_2_O_2_, O_3_, and OH^−^.

Golkowski et al. (2012) studied the in vitro effects of cold plasma (DBD system) on microbial species and found *Staphylococcus aureus* and *Pseudomonas aeruginosa* were considerably (6 log) inactivated following plasma treatment for 1 min. This study demonstrates and stresses the critical role H_2_O_2_ plays in the effectiveness of bacterial inactivation. The research also showed that the bactericidal abilities of free radical effluents might be improved by the presence of H_2_O_2_ investigated the in vitro impact of cold plasma (DBD system) microbial species and reported a significant (6 log) inactivation of the bacterial strains including *S. aureus* and *P. aeruginosa*, after 1 min of plasma treatment. This study indicates and emphasizes the important role played by H_2_O_2_ in bacterial inactivation efficiency. Furthermore, the study revealed that the presence of H_2_O_2_ could also enhance the bactericidal properties of free radical effluents. Cheng et al. (2022) looked into the significance of the main reactive species generated by CAPP during the breakdown of tetracycline hydrochloride. According to degradation routes, O_3_ and H_2_O_2_ were crucial in getting rid of the antibiotic. Similar outcomes were attained when organic contaminants like amoxicillin were removed (Nguyen et al. 2021).

## 7 Essential Oils

Chemically speaking, essential oils (EOs) are volatile hydrocarbons, terpenes, alcohols, aldehydes, and ketones which have a variety of structures and purposes. With the assistance of their chemical components, particularly aromatic compounds, they play a critical role in accelerating free radical stabilization processes in cells (Buonopane, Antonacci, and Lopez 2016; Burt 2004). Based on their diverse structural and functional variations, EOs together possess anti‐inflammatory, antioxidant, antiviral, and anti‐cancer properties. They may work well on their own or in combination with other medications to increase stabilizing effects. The CAPP treatment of EOs has made it possible to easily synthesize them and to increase impact, among other things. In addition, CAPP is employed in conjunction with EOs as a synergistic tool to expand their applicability potential. This is in addition to the extensive area around the CAPP treatment of EOs for multiple applications, such as an increase in EO content, shelf life, wound healing, and antibacterial capabilities. This combination of actions may make it possible to use EOs with lower effectiveness levels while still having a significant impact (Pragna et al. 2019).

In order to ascertain the effect of CAPP (RF plasma) on the antibacterial qualities of EOs, Matan, Nisoa, and Matan (2014) conducted a study. Clove oil, sweet basil oil, and lime oil were the EOs utilized in this study. The major objective of this study was to use CAPP to disinfect chicken eggs from bacterial species using reduced EO concentrations. According to this study, a lower concentration of EOs (5–20 μL mL^−1^) could not cleanse chicken eggs or effectively stop the growth of *E. coli*, *S. typhimurium*, and *S. aureus*. However, after being subjected to CAPP (argon gas, 10 min at 40 W), the concentration of EOs, particularly clove oil (eugenol), substantially suppressed the growth of all these bacterial species. The results of this investigation further demonstrate that although EOs and CAPP can cleanse microorganisms independently (1–3 log_10_), their effective synergy significantly suppressed bacterial growth (6 log_10_).

To evaluate the antibacterial activity of EO alone and after CAPP treatment, experiments on the combined impact of CAPP and EOs (white mustard EO) against bacterial strains (*E. coli*) on meat and lettuce samples were recently conducted. According to this study, white mustard EO and CAPP (5‐min treatment) each have antibacterial characteristics and can prevent the development of bacteria by 1.62 and 2.31 logs, respectively. The same concentration of white mustard EO, however, was able to limit bacterial growth with a 0.48 log reduction following CAPP treatment. This led to the conclusion that CAPP and white mustard EO together have greater antibacterial activity than either compound alone (Qin 2021). The effect of CAPP on the EO content of lemon verbena was presented by Ebadi et al. (2019). The contents and composition of the EO were determined using hydrodistillation and GC/GC–MS. According to this study, a 36% rise in EO content was seen in the CAPP treatment of 1‐min as compared to the control. However, the longer the treatment lasted (up to 5 min), the less significant the increase was in comparison to the control. In terms of composition, oxygenated sesquiterpenes and monoterpene hydrocarbons were found in higher concentrations in CAPP‐treated EO samples, but oxygenated monoterpenes were found in lower concentrations.

The effects of CAPP on the EO content, packaging design, and storage times of hyssop (*Hyssopus officinalis* L.) were investigated by Jangi, Ebadi, and Ayyari (2021). The composition and content of the EO were determined using hydrodistillation and GC/GC–MS. This study demonstrates that the EO content were dramatically reduced after the CAPP treatment (air, *V* = 17–23 kV for 1–10 min) compared to the control, and this reduction was higher for those subjected to CAPP at higher voltages. However, when liquid nitrogen (N_2_) is utilized as a carrier gas, the EO production increases and the EO content barely changes in an N_2_ atmosphere. The effects of CAPP (DBD system) on the essential oil content of turmeric powder were examined by Hemmati et al. (2021). With the aid of GC–MS, the essential oil composition in turmeric powder was both quantitatively and qualitatively determined. It was discovered that the essential oil included 12 distinct chemicals in its entire makeup. Aromatic‐Turmerone, Turmerone, aromatic‐Turmerol, and ‐Sesquiphellandrene were important constituents and CAPP treatment increased the concentration of most of these compounds intermittently.

Yoo et al. (2021) looked into the antibacterial effects of CAPP and clove oil on *S. aureus* and *E. coli* in beef jerky. When CAPP and clove oil were combined, the effects were significantly better than they would have been with either treatment alone (7.0 log reduction vs. 3.0 log reduction, respectively). It became clear from their combination that the effects of CAPP did not result in the degradation of clove oil since they had no effect on the antibacterial activity of clove oil. Cui, Ma, et al. (2016) conducted a comparable experiment on lettuce using CAPP (cold nitrogen plasma) and clove oil. They found that CAPP treatment of EO improved its antibacterial properties. To support the effectiveness of CAPP in decontamination, it was hypothesized that CAPP treated EOs would also manage drug‐resistant microorganisms.

Thyme oil (TO) was used by Lin, Liao, and Cui (2019) to disinfect *S. typhimurium* in poultry meat. This work demonstrates that after TO was treated with CAPP, the antibacterial activity of CAPP‐TO dramatically increased. Following treatment with TO alone and CAPP‐TO, the levels of *S. typhimurium* were determined to be 6.64, 2.24, and 1.15 log CFU g^−1^ at the control, respectively. According to the findings, the packaging sector may find the combination of CAPP and TO to be an appealing replacement due to the longer shelf life of poultry meat. When battling Salmonella enteritidis and *S. typhimurium* on eggshells (below 10 CFU per egg), Cui, Ma, et al. (2016) found comparable results.

Cui et al. (2017) investigated the effectiveness of lemongrass oil in protecting pork loin from *Listeria monocytogenes*. Despite having antimicrobial properties, lemongrass oil needed a high concentration of essential oil, which could harm and lower the quality of pork loin. Lemongrass oil has an antibacterial effect by rupturing bacterial membranes, which causes the breakdown of bacterial cell internal components like DNA and protein. With a lower necessary concentration of essential oil, the synergy of lemongrass oil with CAPP (500 W for 2 min) considerably enhanced the antibacterial action of the essential oil (2.80 log_10_) without sacrificing quality.

It has been extensively studied how to use CAPP to extend the shelf life of food products like beef. The effects of CAPP are increased when used both alone and in conjunction with other natural products. In order to extend the shelf life of chicken breasts, Moradi et al. (2020) looked into the application of CAPP‐treated chitosan and low‐density polyethene bilayer film with summer savory essential oil (SEO). The results showed that applying essential oil assisted by CAPP greatly extended the shelf life of chicken breast (up to 13 days). CAPP and essential oil work together synergistically to extend shelf life, which was previously 6 and 8 days for polyethene and untreated chitosan, respectively. Additionally, the CAPP‐treated‐bilayer film containing SEO can boost the film's tensile strength for extended periods of time while reducing the oxygen transmission rate with less water permeability. The combination effect of *Helichrysum italicum* essential oil and CAPP (cold nitrogen plasma) against *S. aureus* biofilm on food container surfaces was investigated by Cui, Li, et al. (2016). The biofilm was reduced by 4.24 and 2.02 logs, respectively, as a result of the effects of EO and CAPP (CNP, 400 W, 60 s). A larger concentration of EO was needed in addition to the significantly lower reduction power to achieve this reduction. *S. aureus* biofilm was reduced by 5 logs as a result of the combined effects of EO and CAPP.

Sousa et al. (2016) investigated the effects of CAPP on the physiochemical characteristics of cotton and viscose fabrics in the presence of two EOs: *Lavandula angustifolia* (lavender) and *Melaleuca alternifolia* (tea tree). This study uses a variety of analytical and experimental approaches to evaluate surface studies, antibacterial characteristics, and the concentration of hydroxyl and carboxyl groups at the surface. The outcomes supported the synergistic effect of CAPP and EOs in changing the surface characteristics of these textiles. The number of carboxyl groups grew, which enhanced the hydrophobicity and reduced water penetration. When lavender oil was applied to cotton fabric, *S. aureus*'s resistance to infection was increased; however, antibacterial action against *E. coli* was not seen because the minimum inhibitory concentration (MIC) was not reached (Sousa et al. 2016).

Another important research area covers the use of plasma activated oil (PAO) for wound healing (Zou et al. 2019). The study introduces a novel treatment called PAO, which is created using a dry plasma jet process. This process is energy‐efficient, environmentally friendly, and operates at room temperature. The oil used in this study is cheap olive oil, which undergoes a single‐step treatment to enhance its properties for medical use. It has been already observed that the plasma activation process significantly enhances the antimicrobial and antioxidant properties of oils (Yoo et al. 2021). The PAO is produced through a dry plasma‐enabled method that generates reactive species, such as atomic oxygen, which facilitate the oxidation of oil components, leading to increased peroxide and acid values—7.5 times and 57% higher than traditional ozonated oil, respectively. This process not only improves the oil's stability, allowing for a shelf life of up to 1 year, but also enhances its antimicrobial efficacy, as demonstrated by its ability to sterilize wounds and promote faster healing compared to controls. Additionally, the plasma treatment of liquids has shown strong antibacterial effects, effectively inactivating pathogens like *E. coli* and *S. aureus*. The combination of these findings suggests that plasma activation not only improves the physicochemical properties of oils but also significantly boosts their antimicrobial and antioxidant capabilities, making them valuable in medical applications (Schmidt et al. 2019).

## 8 Metal–Organic Frameworks

Metal–organic frameworks (MOFs) are frequently viewed as crystalline porous polymeric materials with superior porous characteristics compared to comparable materials bearing numerous functional groups, resulting in increased tolerability (Li and Huo 2015). Mechanical, ultrasonic, electrochemical, and microwave‐assisted methods are frequently used in MOF synthesis (Stock and Biswas 2012). While making MOFs and composites, however, reaction techniques employed in coordination chemistry as well as for the synthesis of zeolites and porous materials are typically utilized. Synthesis circumstances (such as temperature), activation procedures, the use of a limited amount of solvent with few resulting impurities, high‐yielding techniques, etc. are significant aspects that play a crucial part in their synthesis.

With the expanding range of applications made possible by better synthetic methods, MOFs' diverse functionalities, including their adaptable and tunable porous properties, allow them to be used in gas adsorption, catalysis, sensing, storage, and separation processes. MOF nanoparticles, which are being created with CAPP treatment, are relatively novel materials that belong to the MOFs family; however, further research is needed in this area. In new nano‐catalysts, flexible substrates, innovative adsorbing materials, etc., the synthesis of MOF nanoparticles with desired size and shape, porous characteristics, thermal and chemical stabilities, and greater compatibility is still a hot topic. Due to its ability to function at room temperature, CAPP can be a fantastic tool for treating materials that are sensitive to heat, including polymers, porous organic materials, peptides, proteins, and ultra‐high surface area carbon. (Cogal et al. 2018; Li et al. 2018; Wang et al. 2018).

**FIGURE 8:**
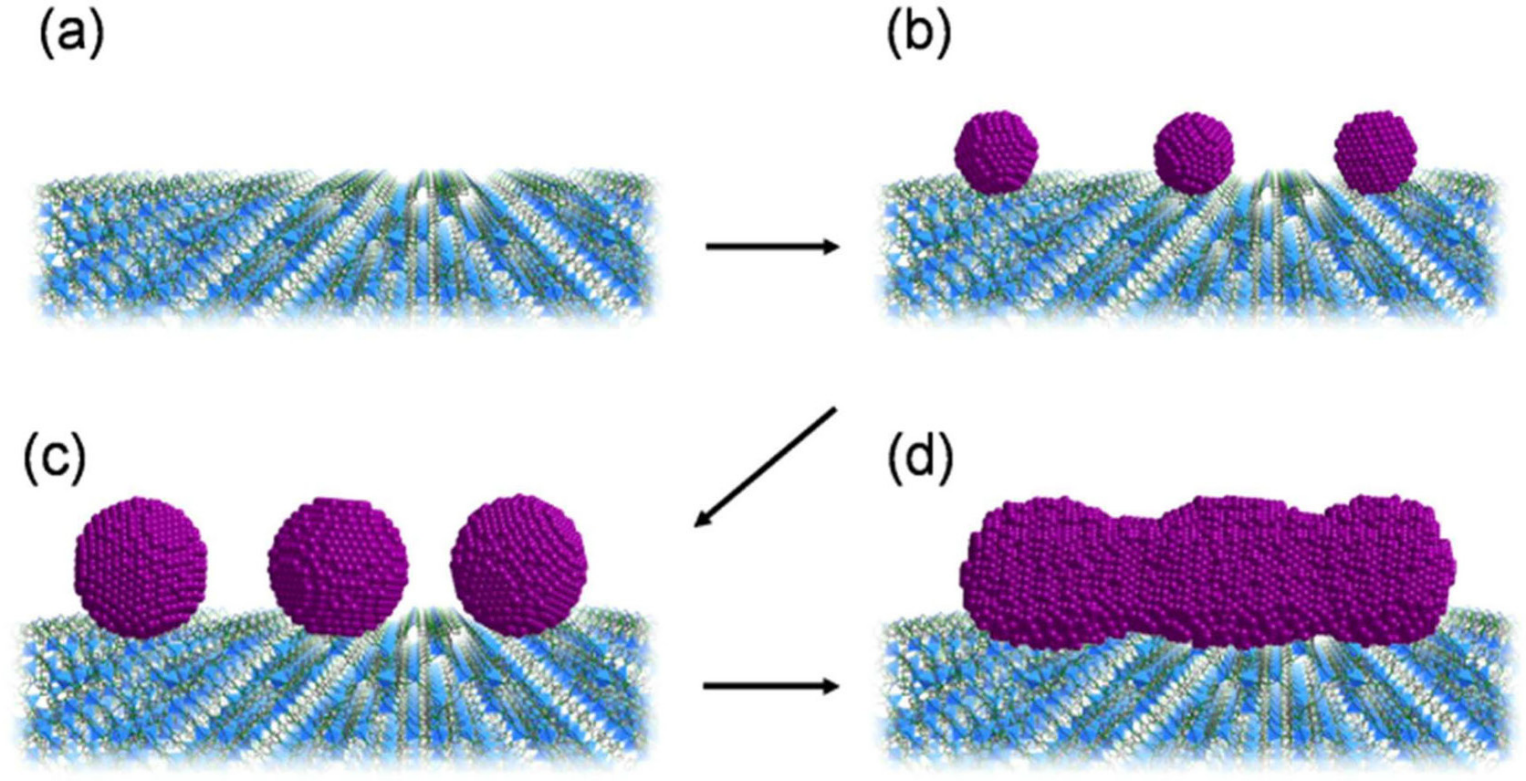
Schematic illustrations of the process of particle formations on the MOFs. (a) Blank MOFs. (b) Formation of seed particles (around 1 nm diameter) at low metal loadings (in the case of Pt, ≈0.5 wt% metal loadings) by APD irradiation. (c) Particle growth up to approximately 2 nm diameter by further APD irradiation (0.5–1.5 wt%). (d) Formation of nanorods by further APD irradiation (above 2 wt%). Reprinted from Sadakiyo et al. (2016).

Arc plasma deposition was employed by Sadakiyo et al. (2016) to synthesize MNPs‐MOFs (Figure 8). For the gram‐scale synthesis of MNP‐MOFs, they used the combination of transition metals Pt, Pd, and Ru on MOF supports like ZIF‐8 and MIL‐101 without utilizing any extra required chemicals or stabilizing agents. The final nanocomposites, Pt/ZIF‐8, pd/ZIF‐8, Ru/ZIF‐8, and Ru/MIL‐101, were 1–2 nm in size. Li et al. (2020) looked into using cold plasma discharge to chemically bond metal oxide nanoparticles, including ZnO, TiO_2_, and iron‐based MOFs, to the surface of a poly(lactic acid) substrate. The photocatalytic capabilities for the breakdown of organic pollutants are improved by this connection. These photocatalysts worked to eliminate ciprofloxacin and photodegrade the rhodamine B dye.

For catalytic CO_2_ methanation, Yanqin et al. (2021) looked into the synergistic effects of CAPP with Co/Zr‐MOF and Co‐nitrate/Zr MOF. The activity of both catalysts was enhanced by CAPP treatment (H_2_–Ar, 13 W, 30 mL min^−1^) for both catalysts. However, CO_2_ methanation was successfully catalyzed by Co/Zr‐MOF under the influence of CAPP. Co/Zr‐MOF and Co‐nitrate/Zr‐MOF converted CO_2_ at a rate of 58.9% and 24.8%, respectively. Co/Zr‐MOF and Co‐nitrate/Zr‐MOF had CH_4_ selectivity that was 94% and 9.8%, respectively, at the same time. Yu et al. (2022) used oxygen plasma for the modification of Ni/Al_2_O_3_ catalyst using Ni‐MOF‐Al_2_O_3_ precursor. The obtained CAPP‐treated catalyst showed better catalytic properties in terms of CO_2_ methanation than other catalysts synthesized from traditional methods. The results showed that the CAPP‐treated nickel‐alumina alloy catalyst was 3.4 times better than other traditional catalysts. Furthermore, according to density functional theory, the activation of CO_2_ was relatively easier for Ni–Al_2_O_3_ than others, whereas Ni–Al_2_O_4_ was unable to activate the CO_2_ for reaction.

## 9 Agricultural Applications

Cold plasma technology has gained attention in agriculture due to its potential applications in various areas. It can be used to modify the surface properties of materials, such as polymers and metals, and has shown promises in inactivating pathogens in the food industry, agriculture, and medicine (Kulaga and Watson 2023). Cold plasma has been used to improve the germinative properties of seeds, with studies showing positive effects on several agronomical specimens (Gao, Francis, and Zhang 2022). The seeds/seedlings have been exposed directly of DBD‐type cold plasma configurations or indirectly soaked in plasma activated liquids (PALs). In both cases, similar microscopic effects have been observed, which include but are not limited to (i) surface modification, (ii) enhanced growth parameters, (iii) higher metabolism, and (iv) boost in disease and stress resistance (Waskow, Howling, and Furno 2021).

### 9.1 CAPP Aided Seed Germination

The ideal seed processing technology should be capable to modifying the seed surface and promoting germination without producing toxins or any detrimental effects on plants or the environment. The cold plasma having low penetration power and associated energy suits well as direct or indirect seed treatment technology. Overall, it has been observed that direct or indirect plasma treatment can accelerate seed germination, reduce water consumption, and most importantly, eliminate microbial or pathogenic contamination with no detectable treatment residues (Măgureanu et al. 2018). It would be quite difficult to state the exact mechanism behind these observations; however, several studies claim the plasma treatments may activate superoxide dismutase, beta‐oxidation, amylases, and proteases and deactivate abscisic acid. For example, Ling et al. (2015) found that non‐thermal plasma treatment enhanced the rapeseed germination under drought conditions. The authors observed that irradiation with cold plasma can cause 15%–20% increase in superoxide dismutase and catalase activities. Besides, seed treatment with cold plasma increased overall sugar and protein contents and reduced the level of malondialdehyde. Some researchers have observed that an increase in seed germination or plant growth might be due to seed surface modifications (preferably erosion) but not due to plant growth harmones (Sidik et al. 2018).

PALs have emerged as a promising indirect activation tool in agricultural science, offering a range of benefits for plant growth and development. Studies have shown that PAW can enhance seed germination, promote plant growth, and improve crop yield (Chalise et al. 2023; Lamichhane et al. 2021; Sivachandiran and Khacef 2017). The application of plasma in agriculture has been linked to increased photosynthesis rates, leading to improved grain quality and yield (Hashizume et al. 2021). Furthermore, PAW has been found to stimulate plant growth by activating the synthesis of plant hormones like auxin and cytokinin, thereby enhancing physiological changes that support plant development (Lamichhane et al. 2021).

CAPP technology has gained traction among agriculturists for its potential to improve crop vitality, production, and sustainability (Adhikari, Adhikari, and Park 2020).

It should be mentioned here that the exact effectiveness of plasma treatment varies with the nature and condition of the seeds to be treated along with the amount of heat energy associated with the plasma irradiation. Hosseini et al. (2018) found that artichoke (*Cynara scolymus* L.) seeds undergo a variety of physicochemical changes and early developments when exposed to cold plasma. reduced modification of seed germination and early development in using low‐energy plasma technology. This study confirms that plasma irradiation can make the seed structure more hydrophilic, which in turn increases the water uptake and accelerates the seed germination. In addition to water or moisture, the temperature or availability of heat exclusively increases the seed germination. In most cases dealing with direct plasma exposure, the temperature of seeds may increase up to 90°C, but this high temperature alone could not be attributed to enhanced seed germination. Actually, the presence of RONS discussed in Section 2.1, especially ozone, NO, and H_2_O_2_, changes seed metabolism and harmone regulation (Waskow, Howling, and Furno 2021). According to Šerá et al. (2021), plasma treatment can reduce the seed hardness and its associated mechanical dormancy.

### 9.2 CAPP for Food Processing and Preservation

Plasma activated liquids (PAL) have shown great promise in decontamination applications, particularly in the food industry and medical settings. Research indicates that liquid plasma immersion with hydrogen peroxide can effectively decontaminate surfaces of pork and chicken meat, offering a potential solution for pathogenic and spoilage bacteria (Sammanee et al. 2022). The antimicrobial properties of non‐thermal plasma and plasma‐activated solutions have drawn attention for their efficacy in controlling and preventing infections, including in hospital settings (Ercan and Ibis 2017).

Plasma technology offers advantages over conventional decontamination methods, as it does not cause corrosion or damage to sensitive equipment, making it suitable for decontaminating surfaces and sensitive materials (Herrmann et al. 1999). The efficiency of plasma liquid decontamination is influenced by various factors such as the type of bacteria, bacterial strain, cell density, and physiological state of the bacteria (Courti et al. 2021). Additionally, the integration of antimicrobial species provided by plasma into non‐thermal treatment modes makes plasma technology attractive for decontamination in food processing and other industries (Schnabel et al. 2019).

The use of low‐temperature atmospheric‐pressure plasmas in the agricultural food production chain has been explored, with a focus on energy efficiency, low carbon emissions, and biodiversity preservation (Judée and Dufour 2020). The CAPP treatment apparatus and design treatment apparatus involve a gas capable of plasma discharge in a gas introduction passage. A pair of plasma generation electrodes downstream along the gas flow passage is used to generate plasma. A second pair of plasma generation electrodes positioned in a second plasma generation position activate the gas species activated in the first plasma region to generate a plasma containing activated gas species. The object to be treated is then exposed to the activated gas species in the second plasma region for treatment. Plasma‐activated liquids or gases can be generated and applied to plants using hand‐held devices, offering a potential method for agricultural use. There are CAPP systems, which consist of feeding cavities, a reaction chamber, an electrode assembly, a high‐frequency power supply, discharging cavities, and a negative pressure device. The reaction chamber has a feed opening and a gas inlet for introducing reaction gas, as well as a discharge opening. A stirring mechanism is arranged in the reaction chamber. The electrode assembly is placed on the outer surface of the reaction chamber and is electrically connected to the high‐frequency power supply. The feeding cavities and discharging cavities are connected to the negative pressure device (Dufour and Gutierrez 2021).

The use of CAPP in raising food output and quality has received a lot of attention in the literature. Additionally, the use of CAPP in decontaminating food against a variety of antimicrobial species has been recommended over other strategies such as chemical sprays, pricey fertilizers, and other products that pose a threat to the environment (Bourke et al. 2018; Gao, Francis, and Zhang 2022). To provide preharvest conditions including growth augmentation, seed sterilization, soil remediation, and post‐harvest food preservation and processing, researchers are implementing CAPP in the agricultural sector (Yan et al. 2022). For example, Kulaga and Watson (2023) used a hand‐held CAPP device that can be directed at different target areas of a plant, and the method involves generating a plasma discharge in a gas or liquid environment and applying it to the plant.

For food processing, many schematic variants of CAPP systems (Table 1) have been used, with microwave‐powered CP systems, plasma jets, and DBD systems being the most popular with various working parameter combinations on various pre‐ and post‐harvest crops.

**TABLE 1:** A brief summary of CAPP production conditions and applications in agricultural and food products.

Plasma system	Plasma parameters	Plant species	Target bacterial/microbial species	Inactivation/decontamination efficiency	References
Microwave‐driven plasma discharge	GFR* = 18 SLM* f* = 2.45 GHz P* = 1.2 kW PT* = 7 s	Pepper seeds, crushed oregano, and paprika powder	Microbial flora	1.6–3 log inactivation	Hertwig et al. (2015)
Microwave‐powered cold plasma treatments (CPTs)	N_2_, He–O_2_ gas mixture (99.8:0.2) at 900 W	Red pepper powder	*Aspergillus flavus* *Bacillus cereus* spores	*A. flavus* = 2.5 ± 0.3 log *B. cereus* = 3.4 ± 0.7 log	Kim, Lee, and Min (2014)
Pulsed light plasma (PLP)	Plasma‐forming gas dry helium *V* = 2.0 kV, *f* = 20 kHz, GFL = 28.0 L min^−1^	Chili flakes	*A. flavus* spores, *Bacillus pumilus* spores, and *Escherichia coli*	1.3 ± 0.1 log spores g^−1^, (*A. flavus*) 2.3 ± 0.2 log spores g^−1^, (*B. pumilus*) > 3.8 log CFU g^−1^ Est. (*E. coli*)	Lee, Park, and Min (2020)
Atmospheric pressure plasma jet, driven at radio frequency	Argon GFR = 20 L min^−1^ into non‐thermal plasma at 10–40 W	Corn leaves	*E. coli* bacteria	3.6 (±0.6) log‐cycle	Baier et al. (2013)
Sinusoidal plasma system	Air gases plasma and sulfur hexafluoride SF6 plasma for up to 20 min *f* = 1 kHz, *V* = 20,000 V *P* = 300 W	Hazelnuts, peanuts, and pistachio nuts	*Aspergillus parasiticus* Total aflatoxins (AFB1, AFB2, AFG1, and AFG2)	1–5 log fungal inactivation 20%–50% aflatoxin reduction	Basaran, Basaran‐Akgul, and Oksuz (2008)
Cold oxygen plasma (COP) system	Atmospheric air *V* = 5–20 kV	Lettuce leaves	*Aeromonas hydrophila*	5.0‐log reduction	Jahid, Han, and Ha (2014)
Microwave‐powered cold plasma	He or a He–O_2_ gas mixture for 2–10 min at 400–900 W	Cherry tomatoes (*Solanum lycopersicum* var. *cerasiforme*)	*Salmonella*	3.5 ± 0.1 log CFU/tomato	Kim and Min (2017)
Plasma jet system	250 W, 15 kHz, ambient air 5–20 min	Brown rice (BR)	*B. cereus*, *B. subtilis*, and *E. coli*	2.30 log CFU g^−1^	Lee et al. (2016)
Dyne‐A‐Mite HP AC pulsed‐plasma jet device	Filtered air, *f* = 47 kHz, *P* = 549 W	Blueberries	Total aerobic plate count (APC) and yeast/molds	0.8–1.6 log CFU g^−1^ 1.5–2.0 log CFU g^−1^	Lacombe et al. (2015)
Microwave‐powered cold plasma	N_2_ and helium–oxygen gas mixture, duration = 1–10 min, *P* = 400–900 W	Cabbage, lettuce, and dried figs	*Salmonella* Typhimurium *Listeria monocytogenes* *E. coli*	0.3–2.1 log CFU g^−1^ (Cabbage) 1.8 ± 0.2 log CFU g^−1^ (Lettuce) 0.5 1.6 log CFU g^−1^ (Dried frigs)	Lee et al. (2015)
Dielectric barrier discharge system	65% O_2_ + 16% N_2_ + 19% CO_2_ and 90% N_2_ + 10% O_2_ *V* = 60 kV, *f* = 50 Hz	Strawberries	Total aerobic mesophiles and yeasts/mold counts	~3.0 log cycles from the initial levels of 5 log_10_ CFU g^−1^	Misra et al. (2014)
Open‐air high‐voltage dielectric barrier discharge (DBD) pilot‐scale reactor	*V* = 115 kV, *P* = 900 W, discharge current (rms average) of 2.2–5.0 A at 2.5‐min plasma exposure	Strawberries and spinach	*E. coli* *Listeria innocua*	2.0 log_10_ CFU mL^−1^ (*E. coli*) 2.2–3.8 log_10_ CFU mL^−1^ (*L. innocua*)	Ziuzina et al. (2020)
Gliding arc cold plasma system	GFL = 10–40 Ls‐160‐Hz AC power Operating outputs of 60 mA at 15 kV	Golden delicious apples	*E. coli* *Salmonella* Stanley	2.9–3.7 log CFU mL^−1^	Niemira and Sites (2008)
Double atmospheric pressure argon cold plasma (DAPACP) jet system	*V* = 25 kV AC sinusoidal wave *f* = ∼25 kHz Argon GFR = 3.5 L min^−1^ Duration = 7.5 min	Date‐palm fruits	*Aspergillus niger*	20 CFU 100 mm^−2^	Ouf, Basher, and Mohamed (2015)
Dielectric barrier discharge system	DC power = 19 V, *I* = 3 A, *f* = 12.5 kHz, GFR = 7 × 10^–3^ m^3^ s^−1^	Fresh cut melon	*Lactobacilli*	5.4–7 LOG CFU g^−1^	Tappi et al. (2016)
Dielectric barrier discharge (DBD)	*V* = 80 kV Duration = 20 min Atmospheric air as carrier gas	Cereal grain (wheat and barley)	Microbiota	2.1–2.4 log_10_ CFU g^−1^ (wheat) 1.5–2.5 log_10_ CFU g^−1^ for bacteria and fungi	Los et al. (2018)

CAPP is used to treat pre‐ and post‐harvested fruits, vegetables, spices, and beverages against diverse microbial strains in agricultural applications. Using the DBD technique, Ali, Cheng, and Sun (2021) looked into how CAPP affected the anilazine fungicide in tomato juice. With 4‐ and 5‐min treatments, CAPP therapy efficiently decomposed anilazine at 47% and 65%, respectively. The evaluation of tomato juice's quality also showed that, other than a slight shift in pH from 4.28 to 4.18, the cold plasma treatment has no appreciable influence on the juice's quality. Furthermore, a minor color change, total flavonoid and phenolic content, and antioxidant content were seen, along with an increase in total soluble solids (5.70–6.70 Brix) and browning index (2.35–4.54). Pankaj, Misra, and Cullen (2013) carried out comparable tests to examine the effect of DBD‐based plasma systems on the tomato peroxidase enzyme. They discovered that plant peroxidase activity was reduced in relation to applied voltage and treatment. The degree of enzyme deactivation increases with applied voltage and treatment time.

### 9.3 Plasma for Microbial Inactivation

The agricultural commodities particularly cereal grains like wheat, corn, rice, and barley, are stored for a long time before their utilization, and risks of microbial contamination are always there. This type of contamination can harm the quality of the commodity and make it unsafe to eat or often spoil, which leads to financial losses for farmers and businesses. CAPP contains RONS, which are very active to destroy bacteria and fungi. A great deal of research has been conducted to check the effectiveness of CAPP technologies and the best ways to use this technology to ensure grains are safe and still good to use.

Los et al. (2018) have tested high‐voltage CAPP on both the natural microorganisms already on the grains and harmful ones that they added for testing. The authors also looked at how the treatment affected the grains' ability to grow into new plants. The study found that after treating barley grains for 20 min, the number of bacteria and fungi was significantly reduced. For example, they measured the reduction in bacteria and found it was about 2.4 log_10_ CFU g^−1^ (which means a lot fewer bacteria). The study found that a specific type of bacteria called *Bacillus atrophaeus* was harder to eliminate, especially its endospores, which are like tough seeds that can survive harsh conditions. However, the cold plasma treatment was still able to reduce their numbers. The effectiveness of the cold plasma treatment varied among different microorganisms. The order of effectiveness was: *E. coli* followed by *P. verrucosum*, then the vegetative cells of *B. atrophaeus*, and finally, the endospores of *B. atrophaeus* were the hardest to kill.

Table 1 describes the effectiveness of cold plasma treatments to destroy or inactivate various types of microbial strains. For example, CAPP configuration having DBD design was used to inactivate bacteria linked to spoilage and pathogenicity in chickens by Rothrock et al. (2017). This study used a DBD‐based cold plasma system to treat liquid cultures of spoilage (*Pseudomonas fluorescens*) and pathogenic bacterial species (*S. enterica typhimurium*, *Campylobacter jejuni*) for treatment times ranging from 30 to 180 s. It was discovered that DBD plasma could inactivate all bacterial species, particularly *C. jejuni*, which was completely destroyed in 30 s (modified air) and 120 s (atmospheric air), while *P. fluorescens* and *S. typhimurium* were destroyed in 180 and 90 s, respectively, using modified air. This suggests that DBD plasma is a potential deactivation and sterilization tool for the poultry industry. In another study, Judée and Dufour (2020) applied a dielectric barrier device to generate cold atmospheric plasma for improving germinative properties. The electrical properties of the resulting plasma have been studied using an equivalent electrical model. The model divides the interelectrode gap into different domains based on the presence of electric charges and the type of medium. By measuring and deducing various parameters, the influence of packed seeds on the plasma electrical properties has been investigated. The model has also been used to assess the total volume of contact points and the capacitance of seeds in the packed‐bed configuration. It has been demonstrated that germinative effects can be induced by plasma on certain agronomical specimens. Overall, cold plasma technology has shown promise as an innovative and effective tool in agriculture, but further research is needed to determine its limitations and negative impacts.

## 10 Conclusions and Future Prospects

Because of its exceptional abilities in antibacterial, sterilizing, and catalyzing reactions involving complexes, surface changes, and pollutant elimination features, cold atmospheric pressure plasma has emerged as one of the most preferred options. Researchers can now employ CAPP in conjunction with other materials, such as composites, polymeric substances, etc., to improve their qualities as synergistic tools of contemporary science and technology. The influence of various plasma components, such as electromagnetic fields and reactive oxygen and nitrogen species, in pre‐harvest and post‐harvest processing has been discussed in detail. The recent advancements in the optimization of parameters of CAPP with varied operating principles have made this possible. The techniques for creating and improving the quality content of essential oils have been changed by the use of CAPP. With the help of CAPP, some MOF features, including improved tolerability, permanent porosity with changeable pore sizes, and nanoparticle production, have been improved. However, creating stable and appealing MOF‐based nanomaterials is a relatively recent field of study that is still being investigated. The comparative treatment of CAPP on pre‐ and post‐harvested agricultural products is still being discussed among research communities, despite the fact that CAPP has already been applied to a variety of agricultural and food products. It should be mentioned here that CAPP technical‐based sterilization was able to reduce the colonies of microorganisms like *B. atrophaeus*, which are harder to eliminate, especially its endospores, are like tough seeds that can survive harsh conditions. This is done to determine whether to treat the agricultural products at the pre‐harvest or post‐harvest level of their production. Similarly, the researchers need to investigate the effects of CAPP treatment on the techno‐functional characteristics of processed foods and the physicochemical properties of protein‐based films. The application of CAPP might be undertaken to reduce the presence of proteins that are responsible for reduction in quality and nutritional value, as well as causing allergic reactions in the human body. Finally, it is worth mentioning here that although EOs and CAPP can cleanse microorganisms independently, the synergetic application of both can surprisingly suppress microbial growth.

## Author Contributions

**Mujahid Ameen Khan:** software (equal), writing – original draft (equal). **Sumia Akram:** writing – review and editing (equal). **Rabia Naeem:** writing – review and editing (equal). **Muhammad Umair Kamal:** methodology (equal), writing – original draft (equal). **Gulzar Muhammad:** writing – review and editing (equal). **Muhammad Mushtaq:** conceptualization (lead), software (lead), supervision (lead), writing – review and editing (lead). **Farooq Anwar:** supervision (lead), writing – review and editing (lead). **Ahmad Hosseini‐Bandegharaei:** supervision (lead), writing – review and editing (equal).

## Conflicts of Interest

The authors declare no conflicts of interest.

## Data Availability

The authors have nothing to report.
